# Dual gene expression cassette vectors with antibiotic selection markers for engineering in *Saccharomyces cerevisiae*

**DOI:** 10.1186/1475-2859-12-96

**Published:** 2013-10-25

**Authors:** Claudia E Vickers, Sarah F Bydder, Yuchan Zhou, Lars K Nielsen

**Affiliations:** 1 Australian Institute for Bioengineering and Nanotechnology, The University of Queensland, Brisbane St Lucia QLD 4072, Australia

**Keywords:** *Saccharomyces cerevisiae*, Expression vector, Antibiotic selection, G418, Phleomycin, Yeast

## Abstract

**Background:**

Manipulations in *Saccharomyces cerevisiae* classically depend on use of auxotrophy selection markers. There are several disadvantages to this in a microbial cell factory setting: (1) auxotrophies must first be engineered in prototrophic strains, and many industrial strains are polyploid/aneuploid prototrophs (2) available strain auxotrophies must be paired with available repair plasmids (3) remaining auxotrophies must be repaired prior to development of industrial bioprocesses. Use of dominant antibiotic resistance markers can circumvent these problems. However, there are relatively few yeast antibiotic resistance marker vectors available; furthermore, available vectors contain only one expression cassette, and it is often desirable to introduce more than one gene at a time.

**Results:**

To overcome these problems, eight new shuttle vectors have been developed. The plasmids are maintained in yeast under a 2 μm *ori* and in *E. coli* by a pUC *ori*. They contain two yeast expression cassettes driven by either (1) the constitutive *TEF1* and *PGK1* promoters, or (2) the constitutive *TEF1* promoter and the inducible *GAL10* or *HXT7* promoters. Expression strength of these promoters over a typical production time frame in glucose/galactose medium was examined, and identified the *TEF1* and *HXT7* promoters as preferred promoters over long term fermentations. Selection is provided by either *aphA1* (conferring resistance to G418 in yeast and kanamycin/neomycin in *E. coli*) or *ble* (conferring resistance to phleomycin in both yeast and *E. coli*). Selection conditions for these plasmids/antibiotics in defined media were examined, and selection considerations are reviewed. In particular, medium pH has a strong effect on both G418 and phleomycin selection.

**Conclusions:**

These vectors allow manipulations in prototrophic yeast strains with expression of two gene cassettes per plasmid, and will be particularly useful for metabolic engineering applications. The vector set expands the (currently limited) selection of antibiotic marker plasmids available for use in yeast, and in addition makes available dual gene expression cassettes on individual plasmids using antibiotic selection. The resistance gene cassettes are flanked by *loxP* recognition sites to allow CreA-mediated marker removal and recycling, providing the potential for genomic integration of multiple genes. Guidelines for selection using G418 and phleomycin are provided.

## Background

*Saccharomyces cerevisiae* is a model organism and an industrial workhorse. It is readily transformed with plasmid expression vectors, and is therefore particularly amenable to functional analysis experiments and genetic engineering applications. Plasmid vectors may be integrative (yeast integrating plasmids, YIp), autonomously replicating high copy-number vectors (yeast episomal plasmids, YEp), or autonomously replicating low copy-number vectors (yeast centromeric plasmids, YCp) [1,2]. Typically, selection for presence/genomic integration of the plasmid is performed by complementation/repair of a chromosomal mutation resulting in auxotrophy for an amino acid. Commonly-used markers include *URA3, HIS3, LEU2, TRP1* and *LYS2*. A wide variety of yeast strains containing one or several mutations with low reversion rates conferring appropriate auxotrophies are available (e.g. *ura3-52, his3-Δ1, leu2-Δ1, trp1-Δ1* and *lys2-201*[1]; see the *Saccharomyces* Genome Database, http://www.yeastgenome.org/). However, to use these strains one must have a plasmid bearing an appropriate auxotrophy gene, and vice-versa: the plasmid bearing a particular gene of interest must encode an auxotrophy repair gene for which the cognate auxotrophy is found in the strain of interest. This may not be the case, particularly in heavily engineered strains where more genes are being introduced, as auxotrophies may already be complemented/repaired. Furthermore, any remaining auxotrophies must be repaired in engineered strains for which industrial bioprocesses are being developed (e.g. [3]). In addition, wild-type and industrial strains are typically polyploid or aneuploid prototrophs [4]. Engineering in prototrophs requires use of dominant markers and/or time-consuming construction of auxotrophy mutants, which may be difficult or impossible in a complex genetic background.

An alternative to using auxotrophies is to use dominant resistance markers. Selection genes conferring resistance to various substances are available (reviewed in [2,5]). Among these, resistance to antibiotics is most commonly used. The antibiotics G418 [6-10], hygromycin B [8-14], phleomycin [15], chloramphenicol [16], nourseothricin [8,13,14], bialaphos [13], zeocin [9], glufosinate [9] and aureobasidin A [17] can be used for selection of *S. cerevisiae* transformed with appropriate resistance genes, and shuttle vectors conferring resistance to several of these antibiotics (or equivalents) in *E. coli* have also been developed. However, the yeast community has historically used auxotrophy repair/complementation more widely than antibiotic selection because of the insensitivity of yeast to many antibiotics and the frequency of spontaneous resistant mutants [18]. Furthermore, the efficiency of transformation and transformant recovery is often lower using antibiotic marker selection. Nowadays, antibiotic resistance vectors that provide improved transformant selection are available (e.g. [19]), and greatly improved yeast transformation methods (e.g., [20]) as well as improved antibiotic selection methods (e.g. [21]) mean than efficiencies similar to those obtained using auxotrophy selection can be achieved.

Current antibiotic selection plasmids typically contain only one gene expression cassette. However, it is often desirable to introduce more than one gene at a time, particularly for metabolic engineering projects where multiple modifications and/or complex pathway reconstruction is required. Several dual gene expression cassette vectors have been developed [22-26], as have single gene expression vectors that can be used combinatorially for metabolic engineering applications [27]. However, all of these vectors rely on auxotrophy selection markers. Currently, to our knowledge there are no dual expression vector plasmids with antibiotic selection available. Here, we describe development of dual gene expression shuttle vectors for yeast transformation/engineering using dominant antibiotic marker genes. Antibiotic selection characteristics of *S. cerevisiae* strains bearing these plasmids were examined, and guidelines for antibiotic use in defined media are provided.

## Results and discussion

### Vector construction and promoter analysis

Partow et al. (2010) recently developed a pair of dual cassette expression vectors for use in glucose-containing media. They replaced the bi-directional galactose-inducible *GAL1-GAL10* promoter region in pESC-Ura (Stratagene; now supplied by Agilent Technologies http://www.genomics.agilent.com) with a *TEF1-PGK1* bi-directional promoter developed from the transcriptional elongation factor EF-1α (*TEF1*) and phosphoglycerate kinase (*PGK1*) promoters. These glycolysis gene promoters perform well on glucose-based media in *S. cerevisiae* CEN.PK-derived strains [22]. Two vectors (pSP-G1 and pSP-G2) were constructed, with the bi-directional promoter in both orientations relative to the multiple cloning site and terminator sequences (either *ADH1* or *CYC1* terminators from *S. cerevisiae*) [22].

**Figure 1:**
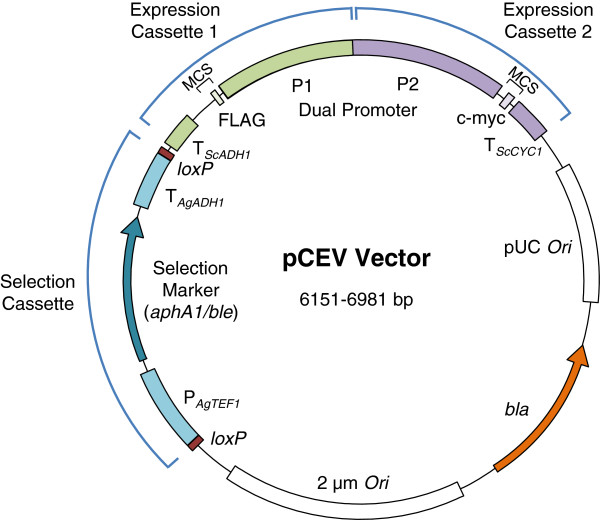
**Generic plasmid vector map for the pCEV dual gene cassette expression vectors.** Each plasmid has two expression cassettes, each driven by a different promoter (P1 or P2), and a selection cassette with either *aphA1* or *ble* genes. Promoter/selection combinations are shown in Figure 1. Each gene of interest can be optionally tagged using either a FLAG epitope tag (Expression Cassette 1) or a c-myc epitope tag (Expression Cassette 2). The selection cassette is controlled by the *TEF* promoter and terminator from *Ashbya gossypii* (P_*AgTEF1*_ and T_*AgTEF1*_). The terminator for Expression Cassette 1 is derived from the yeast alcohol dehydrogenase (*AHD1*) gene. The terminator for Expression Cassette 2 is derived from the yeast cytochrome C (*CYC1*) gene. The potential exists for integration of the expression cassettes onto the chromosome by amplification using primers with homologous arms; in this case, the selection marker cassette can be removed to recycle the marker via CRE-mediated recombination at the available *loxP* sites. A pUC origin of replication (*Ori*) is available for maintenance in *E. coli* and a 2 μm *Ori* for maintenance in *S. cerevisiae*. Selection in *E. coli* can be performed using either the *aphA1/ble* selection cassette, which is functional for selection in *E. coli*, or using the beta-lactamase (*bla*) ampicillin resistance gene. The dual promoter region is not to scale (size varies depending on which promoters are present) and the selection marker is also not to scale (can be either *aphA1* or *ble*). Different vector components in these two regions, as well as the unique restriction enzyme sites found in each multiple cloning site (MCS), are shown in Table 2. Note that the MCS spans the epitope tag sites for each expression cassette; choice of restriction sites will determine whether or not the tag sequence is retained in the construct.

To develop dual gene expression shuttle vectors for yeast transformation/engineering with dominant antibiotic marker genes, we replaced the uracil auxotrophy selection marker (*URA3*) in pSP-G1 and pSP-G2 with either *aphA1* or *ble* cassettes flanked by *loxP* recombination sites from pUG6 or pUG66 [28-30] respectively. The *aphA1* gene confers resistance to kanamycin (in *E. coli*) and G418 (in yeast); the *ble* gene confers resistance to phleomycin in both *E. coli* and yeast. Gene replacement resulted in four different plasmids with all permutations of *TEF1-PGK1* promoter orientation and resistance gene: pCEV-G1-Ph, pCEV-G1-Km, pCEV-G2-Ph and pCEV-G2-Km (Figure 1, Table 1).

**Table 1:** Details for plasmid vectors

**pCEV vector plasmid**	**Expression cassette 1**		**Expression cassette 2**		**Selection marker**	**GenBank accession**
	**Promoter**	**Unique RE sites**	**Promoter**	**Unique RE Sites**		
pCEV-G1-Km	TEF1	NotI, SpeI, PacI	PGK1	BamHI, ApaI, ApaI, XmaI, SalI, Acc65I, KnpI, SacII, NheI	aphA1	KF366478
pCEV-G1-Ph	TEF1	NotI, SpeI, PacI	PGK1	BamHI, ApaI, HindIII, Acc65I, KnpI, SacII, NheI	ble	KF366479
pCEV-G2-Km	PGK1	NotI, SpeI, PacI	TEF1	BamHI, ApaI, ApaI, XmaI, SalI, Acc65I, KnpI, SacII, NheI	aphA1	KF366480
pCEV-G2-Ph	PGK1	NotI, SpeI, PacI	TEF1	BamHI, ApaI, HindIII, Acc65I, KnpI, SacII, NheI	ble	KF154123
pCEV-G3-Km	GAL10	SpeI, PacI	TEF1	BamHI, ApaI, ApaI, XmaI, SalI, Acc65I, KnpI, SacII, NheI	aphA1	KF366481
pCEV-G3-Ph	GAL10	SpeI, ClaI, PacI	TEF1	BamHI, ApaI, HindIII, Acc65I, KnpI, SacII, NheI	ble	KF366482
pCEV-G4-Km	HXT7	SpeI, PacI	TEF1	BamHI, ApaI, XmaI, SalI, Acc65I, KnpI, SacII, NheI	aphA1	KF366483
pCEV-G4-Ph	HXT7	SpeI, ClaI, PacI	TEF1	BamHI, HindIII, Acc65I, KnpI, SacII, NheI	ble	KF366484

Unique restriction sites are shown for commonly-found restriction enzymes (RE) in the multiple cloning site for each expression cassette (other unique sites are also present). Plasmid datasheets are available in the Additional file 1. Plasmid maps in Clone Manager format (Sci-Ed Software, http://www.scied.com) are available through the AddGene website (http://www.addgene.org/).

In contrast to Partow et al. (2010), who examined promoter strength in CEN.PK-derived strains, we observed unexpectedly poor expression strength when using the *PGK1* expression cassette in our vectors with S288C-derived strains (data not shown). We therefore examined the relative strength of several promoters using a β-galactosidase reporter gene assay in an S288C-derived strain. As well as the *TEF1* and *PGK1* promoters, we included the *GAL10* promoter, since the S288C-derived strain EPY210C [31] that we were using also has galactose-inducible modifications, and we were interested in using this promoter for future engineering experiments. In addition, we tested a high affinity hexose transporter (*HXT7)* promoter [22]. The *HXT7* gene is up-regulated when glucose concentrations are low and repressed when glucose concentrations are high [22,32-34], and therefore provides a potential alternative for driving gene expression in media with low/no glucose - including galactose or glucose/galactose medium used for *GAL* promoter-driven expression (e.g., [3,35-37]). Available promoter:β-galactosidase yeast integrating plasmids were used for the *TEF1* and *PGK1* promoters [22], and similar plasmids were constructed for the *HXT7* and *GAL10* promoters (see Methods).

**Figure 2:**
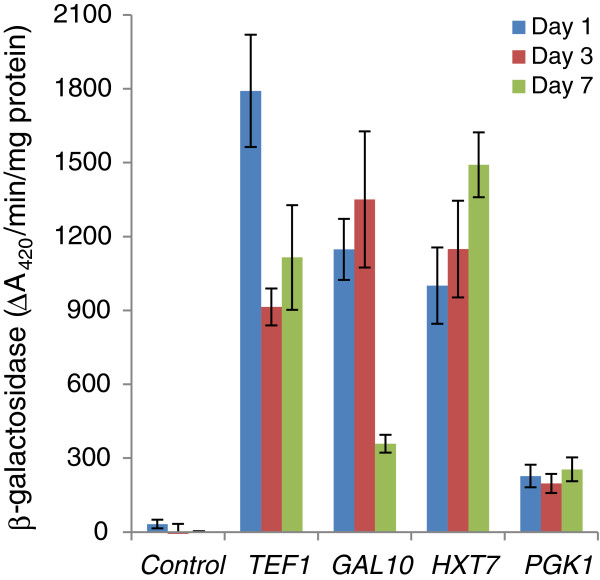
**β-Galactosidase activity driven by various promoters in the *S. cerevisiae *S288C-derived strain EPY210C growing on galactose.** EPY210C [31] was used as a base for strain construction to test promoter expression strength using promoter:*lacZ* constructs on integrative plasmids. Plasmids pSF015 (*HXT7* promoter), pSF016 (*PGK1* promoter), and pSF019 (*TEF1* promoter), bearing promoters amplified from *S. cerevisiae* CEN.PK, have been described previously [22]. We reconstructed a P_*GAL10*_: *lacZ* fusion construct (pGAL10lac) by amplifying the relevant region from *S. cerevisiae* S288C genomic DNA (see Methods). The negative control was the promoterless *lacZ* plasmid pSF011 [22]. β-Galactosidase assays are described in the Methods. Bars are means of n = 3 biological replications; errors are standard deviations.

Reporter gene activity driven by the *TEF1*, *PGK1, GAL10* and *HXT7* promoters was examined over several days in cultures grown on rich medium containing 0.2% glucose + 1.8% galactose (Figure 2). This sugar combination is used to build up biomass prior to inducing expression of desired genes (e.g. [3,35,37]). In this medium, glucose is rapidly consumed within the first 6–8 hours of fermentation; growth then shifts to being galactose-based. This experiment confirmed that the *PGK1* promoter drives relatively poor expression in *S. cerevisiae* S288C-derived strains growing on galactose. Over the short term (24 hr, by which time cultures are in stationary phase), the *TEF1* promoter emerged as the strongest promoter, with the *GAL10* and *HXT7* promoters driving slightly lower, but similar levels of expression. By the third day, β-galactosidase activity was similar for the *TEF1*, *GAL10* and *HXT7* promoters. By day 7, the *TEF1* and *HXT7* promoters were still driving good levels of expression, while β-galactosidase activity driven by the *GAL10* promoter was much weaker. Depending on the time scale of the fermentation and relative stability of the gene products, both the *GAL10* and *HXT7* promoters show utility for glucose/galactose fermentations using S288C-derived strains. We therefore replaced the weak *PGK1* promoter with *GAL10* or *HXT7* promoters. This resulted in four new dual gene expression plasmids (Table 1, Figure 1). In plasmids containing the *GAL10*-controlled expression cassette (pCEV-G3-Km and pCEV-G3-Ph), the potential exists to generate a third expression cassette by inserting an MCS and a terminator sequence between the bidirectional *GAL10-1* promoter region and the *TEF1* promoter. This third expression cassette would be controlled by the *GAL1* promoter. Genes already fused to a terminator can be placed under the control of the *GAL1* promoter by insertion through the unique FseI restriction site in pCEV-G3-Ph, or the unique FseI, KroI, NaeI or NgoMIV restriction sites in pCEV-G3-Km.

### Selection characteristics on G418 and phleomycin

In the process of applying the expression vectors for yeast engineering projects, we learned a number of things about using G418 and as selective agents. In particular, we observed plasmid instability and poor selection on defined media. For all 2 μ plasmids, we observed spontaneous plasmid loss after 48 hr in non-selective medium (data not shown); we have previously observed this for 2 μ auxotrophy selection plasmids as well [31]. We have summarized our observations, along with a review of the relevant literature, in the sections below. In addition, we did a detailed study on the effects of pH on selection.

#### ***G418***

The *aphA1* gene from the *E. coli* transposon Tn*903*[38] encodes an aminoglycoside 3′-phosphotransferase activity which confers resistance against G418 (sold as Geneticin® by Invitrogen/Life Technologies) in *S. cerevisiae* and kanamycin in *E. coli*[4,6,18,39]. It is also referred to as *kan* (for kanamycin resistance), *neo* (for neomycin resistance) or *npt1* (for neomycin phosphotransferase). G418 and kanamycin are 2-deoxystreptamine aminoglycoside antibiotics; they act by inhibition of protein translation at the elongation step [40]. The level of resistance conferred depends on several factors, including expression strength and copy number. The *kanMX* module [39] contains *aphA1* (*kan*) under the control of promoter and terminator sequences from the *TEF* gene of the filamentous fungus *Ashbya gossypii*. It confers resistance to G418 at 200 μg/mL in *S. cerevisiae* grown on YPD (YEPD) and kanamycin at 30 μg/mL in *E. coli* grown on LB. This cassette was subsequently flanked by *loxP* sites to allow facile marker gene removal during knock-out and tagging experiments via the CRE-*loxP* recombination system [28]. The *kanMX loxP* construct was used in the current work for plasmid assembly.

Medium composition affects the efficiency of G418 selection [18]. In particular, pH has a strong effect on aminoglycoside phosphotransferase activity [41]; therefore, medium pH may modulate selective pressure. Activity is higher at pH 7 than at decreased pH such is found in typical defined yeast media (often around 5.5; [42,43]). While selection in complex YPD medium is effective at 200 μg/mL G418 in YPD, we encountered problems when using defined media. To examine how pH affects selective power under these conditions, we tested growth on defined medium with the pH adjusted to either 5 or 7 and supplemented with a range of G418 concentrations (Table 2). At both pH 5 and pH 7, selection was ineffective at 200 μg/mL G418. Effective selection could be achieved at 400 μg/mL G418 at both pH values. This indicates that the aminoglycoside transferase activity was in fact similar under both pH conditions, and that some other medium component/condition affects selectivity at concentrations below 400 μg/mL G418. In addition to pH, ammonium sulphate is known to interfere with G418 selection; it can be replaced with monosodium glutamate at 1 g/L to improve selection characteristics without modifying medium pH [44].

**Table 2:** Antibiotic sensitivity testing

**pH**	**Strain**	**G418 (μg/mL)**				**Phleomycin (μg/mL)**				
		**200**	**400**	**600**	**800**	**20**	**40**	**60**	**80**	**100**
5	S288C	+++	-	-	-	+++	+++	++	++	+
	EPY210C(pCEV-G2-Ph)	+++	-	-	-	+++	+++	+++	+++	+++
	EPY201C(pCEV-G2-Km)	+++	+++	+++	+++	+++	+++	++	++	+
7	S288C	+++	-	-	NT	++	-	-	NT	NT
	EPY210C(pCEV-G2-Ph)	+++	-	-	NT	+++	+++	+++	NT	NT
	EPY201C(pCEV-G2-Km)	+++	+++	+++	NT	++	-	-	NT	NT

Strains were streaked onto SD medium with pH adjusted to either 5 or 7, and supplemented with antibiotics at the concentrations noted. +++, strong growth, large colonies; ++, slow growth, small colonies; +, very poor growth in primary inoculum region only, no defined colonies; -, no growth. NT = not tested.

It should also be noted that G418 efficiency can decrease at high cell densities in mammalian cells [45]; this can presumably occur in yeast as well. In addition, commercial G418 preparations can vary in purity and potency, even in between batches [28]. In summary, the following considerations should be taken into account when using G418 as a selective agent:

1. Medium pH should be considered, as aminoglycoside transferase activity decreases with decreasing pH.

2. Medium composition affects selection conditions.

3. Selection conditions may vary with strain.

4. Loss of G418 resistance may occur over long incubation periods; this is most likely related to high culture density. Dosing in more antibiotic may improve selection over long cultivations to prevent plasmid loss.

5. Plating density may also affect selection.

6. Growth rates may decrease in the presence of G418.

7. Adjusting the pH to neutral or increasing antibiotic concentrations may be required.

#### ***Phleomycin***

The *ble* gene from the bacterial transposon Tn*5* confers resistance to the metallo-glycopeptide class antibiotics bleomycin and phleomycin in both prokaryotes and eukaryotes [15,46-48]. These antibiotics act by binding to DNA and causing strand breakage [49-51]. Efficient DNA binding and breakage requires metal ions (ferrous ions) and molecular oxygen as cofactors [50,51]. Accordingly, *S. cerevisiae* is significantly more resistant to phleomycin under anaerobic conditions [15], and presence of EDTA decreases/removes toxicity [52]. Sensitivity to phleomycin depends on growth phase in *S. cerevisiae*: stationary phase cells are less sensitive than exponentially growing cells [52].

The Ble protein inactivates phleomycin family antibiotics by binding to them [53]. The *ble* gene was first developed as a selective marker for phleomycin resistance in *S. cerevisiae* by Gatignol et al. [15]. The *aphA1* gene in the *kanMX* module was replaced by the *ble* gene to develop a second *TEF1* promoter-driven dominant marker gene knock-out cassette [29]. This construct conferred resistance to 7.5 μg/mL phleomycin in *S. cerevisiae* (CEN.PK2-1C) growing on YPD. In early studies, incubation in non-selective medium prior to application of selection pressure was required to obtain transformed cells with reasonable efficiency [21], though Gueldener et al. [29] later found that pre-incubation did not enhance transformation efficiency. This may be due to genotypic differences between yeast strains (OL1 in the former study and CEN.PK-derived in the latter study), differences in expression characteristics (*CYC1* promoter in the former vs. *TEF1* in the latter) and/or differences in growth media. In our hands, we found that a three hour incubation in non-selective medium is optimal for efficient transformation in the S288C-derived strain EPY210C being selected on YPD (data not shown). Plating at high densities can also decrease transformation efficiency [21].

Most commercial preparations of phleomycin suggest using 10 μg/mL for selection of *S. cerevisiae* on YPD, as determined by Gatignol et al. [19]. However, we observed colonies of S288C-derived strains (without plasmid) on YPD plates containing phleomycin at 10 μg/mL after three days (data not shown). This suggests that spontaneous resistance can develop over that timeframe under those conditions. Increasing the concentration to 15 μg/mL inhibited growth significantly over three days; using 20 μg/mL completely inhibited development of spontaneous resistance over a seven day period (data not shown).

Phleomycin sensitivity decreases at lower pH values [54], such as commonly found in defined yeast media [42,43]. We therefore examined selection efficiency in defined medium at pH 5 and 7, again using several different concentrations of phleomycin (Table 2). Selection was essentially ineffective at pH 5. Some selective pressure was observed at 60–80 µg/mL; selective pressure increased significantly at 100 µg/mL, but growth was not completely repressed in control strains even at 100 µg/mL. When the pH was adjusted to 7, selection was highly effective at concentrations at or above 40 µg/mL.

We also observed that the *TEF1* promoter-driven *ble* module could confer resistance to phleomycin in *E. coli* (data not shown), as shown for other yeast *ble* constructs [15]. This allows dual antibiotic selection of transformed *E. coli* (using ampicillin at 100 μg/mL and phleomycin at 5 μg/mL).

In summary, the following considerations should be taken into account when using phleomycin as a selective agent:

1. Phleomycin activity requires the presence of molecular oxygen and ferrous ions. Anaerobic fermentations may require significantly higher phleomyin concentrations.

2. Plating at high densities can decrease transformation efficiency.

3. Depending on strain/medium, efficient selection may require incubation on non-selective medium prior to application of selective pressure, and may also affect maintenance of selection.

4. Sensitivity to phleomycin decreases below neutral pH. Phleomycin concentration may need to be increased significantly in media with lower pH values; ideally, medium pH should be adjusted to 7.

5. Antibiotic titration/dosing may be required during prolonged cultivations to prevent development of spontaneous resistance and/or plasmid loss.

6. Stationary phase cells are less sensitive to phleomycin than exponentially growing cells.

## Conclusions

In summary, we have developed dominant marker plasmids containing two gene expression cassettes that provide selection in *Saccharomyces cerevisiae* via either phleomycin or G418 resistance. A caveat of using G418 and phleomycin for selection is the requirement for adjustment of medium pH to neutral and/or increasing antibiotic concentration to maintain selective pressure on defined media. Expression of genes in each plasmid can be controlled by a combination of the *TEF1* promoter with either the *PGK1*, *GAL10* or *HXT7* promoters. Each promoter is followed by a multiple cloning site (MCS) and a transcription termination sequence. In addition, both expression cassettes encode epitope tags that can be used for C- or N-terminal tagging. The plasmids are shuttle plasmids with a 2 μm *ori* (yeast) and a pUC *ori* (*E. coli*). Selection in *E. coli* is conferred by a beta-lactamase (*bla*) ampicillin resistance gene as well as the *aphA1* or *ble* genes. The *TEF1*, *GAL10* and HXT7 promoters drive strong expression in galactose medium; *TEF1* and *HXT7* are preferred for high expression over prolonged fermentations. The *HXT7* promoter might also be useful in other non-/low-glucose media (e.g. sucrose-based media). We have subsequently used these plasmids successfully for expression of isoprenoid production genes in yeast (data not shown). Genomic integration of the expression and selection cassettes could be readily achieved by amplification using primers with suitable homologous arms [55]. PCR-mediated integration of similarly large fragments has been demonstrated recently, and useful integration loci have been characterized [23,27]. The presence of the *loxP* sites allows removal of selective marker genes by CreA-mediated recombination so they can be re-used for subsequent modifications [28,55]. The *aphA1* or *ble* genes can also be replaced by genes encoding alternative selection functions (e.g., see review in Introduction) to expand the available selection systems. The antibiotic marker vectors can also be used to pyramid further modifications into strains where available auxotrophy markers have been exhausted by insertion of multiple constructs. In addition, they may also be useful in other yeasts and filamentous fungi that are sensitive to phleomycin or G418; for example, the yeasts *Schizosaccharomyces pombe*[56] and *Cryptococcus neoformans*[57] and the filamentous fungi *Aspergillus flavus*[54], *Neurospora crassa*[58], *Aspergillus nidulans*[58] and *Pleurotus ostreatus*[59] are all sensitive to phleomycin. The plasmids are available through AddGene (http://www.addgene.org/) and Euroscarf (http://web.uni-frankfurt.de/fb15/mikro/euroscarf/). Detailed plasmid datasheets are available in Additional file 1 and annotated sequence files in Clone Manager format (Sci-Ed Software, http://www.scied.com) are available through the AddGene website (http://www.addgene.org/). Sequence data can also be downloaded from GenBank using the provided accession numbers (Table 1).

## Methods

### Strains and media

*E. coli* strain MG1655 (Coli Genetic Stock Centre CGSC#7740, New Haven CT, USA) or α-select (Bioline; Alexandria NSW, Australia) were used for plasmid manipulations; they were maintained on LB medium [60], and transformations were performed by chemical transformation according to the manufacturer’s instructions. *S. cerevisiae* S288C was kindly provided by the Australian Wine Research Organisation, and was originally sourced from the American Type Culture Collection (http://www.atcc.org/; ATCC Accession: 204508). The S288C-derived *S. cerevisiae* strain EPY210C [31] was used as a base for strain construction to test promoter expression strength (see below). *S. cerevisiae* strains were grown on YPD [43] for general growth and selection, and on YPD with the glucose replaced with 0.2% glucose and 1.8% galactose (YPDG) for promoter analysis experiments (see below). To examine the effect of pH on selective power in defined medium, yeast were grown in SD medium [43] with the pH adjusted to either 5 or 7 (using potassium hydroxide) and filter-sterilised. All media were supplemented with appropriate antibiotics at the concentrations stated. Antibiotics G418 sulfate and Phleomycin were sourced from Invivogen (San Diego, California; cat. # ant-gn-5 and ant-ph-5, respectively).

### Plasmids and plasmid construction

The uracil auxotrophy selection marker in pSP-G1 and pSP-G2 [22] was replaced with either *aphA1* or *ble* cassettes containing *loxP* recombination sites from pUG6 or pUG66 [28,29] (sourced from Euroscarf), respectively. pUG6 and pUG66 were prepared for use as PCR templates by digesting with BglI. The digested plasmids were used as PCR templates with primers HA1-kanphleo and HA2-kanphleo (Table 3). For HA1-kanphleo, the 5′ sequence is homologous with sequence just downstream from the 2 μm ori pUG6/pUG66. The SalI site in pUG6 is destroyed, but the AccI site (underlined) remains for facile removal of the cassette. For HA2-kanphleo, the 5′ sequence is homologous with sequence at the end of the *ADH1* terminator in pUG6/pUG66. The sequences in italics are the priming sites in pUG6/pUG66. Resulting PCR products have arms that are homologous to sequences upstream and downstream of the URA marker in pSP-G1 and pSP-G2; replacement of the URA3 gene with the *kanMX* or *ble* cassettes removes the F1 ori in pSP-G1/pSP-G2. PCR products were digested with DpnI. The vectors (pSP-G1/pSP-G2) were prepared by digestion with EcoRV and NcoI and then cleaned. Fragments were transformed into electro-competent *E. coli* MG1655 cells harbouring the λ Red recombinase plasmid pKD46 [61] for cloning by homologous recombination ('recombineering’; [62,63]). Selection was performed on ampicillin (100 μg/mL) plus either kanamycin (30 μg/mL) or phleomycin (5 μg/mL). Clones were screened by colony PCR using primers 2muDown and ADH1-T F1 (Table 3). Plasmids isolated from positive clones were screened by restriction analysis and the insert fully sequenced using the same primers. Resulting plasmids were pCEV-G1-Ph, pCEV-G1-Km, pCEV-G2-Ph and pCEV-G2-Km.

**Table 3:** Primer sequences used in this study

**Primer**	**Sequence**	**Application/notes**
HA1-kanphleo	ATGCTATCATTTCCTTTGATATTGGATCATGGTA*GACAACCCTTAATATAACTTCGTA*)	Cloning of antibiotic resistance gene cassettes. Sequences in italics are priming sites in pUG6/pUG66. For HA1-kanphleo, the 5′ sequence is homologous with sequence just downstream from the 2 μm ori pSPG1/pSPG2; the SalI site in pUG6 is destroyed, but the AccI site (underlined) remains for facile removal of the cassette. For HA2-kanphleo, the 5′ sequence is homologous with sequence at the end of the *ADH1* terminator in pSPG1/pSPG2.
HA2-kanphleo	TGCTTTCTCAGGTATAGCATGAGGTCGCTC*CTAGTGGATCTGATATCACC*	
2muDown	CCATTCCATGCGGGGTATCG	Screening colonies and sequencing for pCEV-G1-Ph, pCEV-G1-Km, pCEV-G2-Ph and pCEV-G2-Km
ADH1-T F1	TCGTTGGTAGATACGTTGTTGAC	
GAL10LacF	*TGATATCGAATTCCTGCAGCCCGGG*GGATCCGTTTTTTCTCCTTGACGTTAAAGTA	Amplification of *GAL10-GAL1* promoter region from *S. cerevisiae* S288C. The sequence in *italics* is homologous to the target vector pSF011 [22]. Restriction enzyme sites are underlined. The remaining sequence is the priming site for *S. cerevisiae* S288C genomic DNA.
Gal10LacR	*GTAATCATGGTCATGGTGCGGCCG*CTCTAGAGAATTTTCAAAAATTCTTACTTTTTTTTTG	
GAL10P2B	GTGTGCGGCCGGCC*GTTTTTTCTCCTTGACGTTAAAGTA*	Amplification of the *GAL* promoter region from pGAL10lac. Restriction sites are underlined and priming sequence is italicised
GAL10P1A	GATCCCACTAGT*GAATTTTCAAAAATTCTTACTTTTTTTTTG*	
HXT7P1B	GTGTGCGGCCGGCC*CCGTGGAAATGAGGGGTATG*	Amplification of the *HXT7* promoter from pSF015 [22]. Restriction sites are underlined and priming sequence is italicised
HXT7P2A	GATCCCACTAGT*TTTTTGATTAAAATTAAAAAAAC*	
CYC1-TR1	GGGACCTAGACTTCAGGTTGTC	Screening for promoter:*lacZ* yeast strains
Lac9434r	GAAGCCTGCGATGTCGGTTTC	
ADH1-T R1	GGAGCGACCTCATGCTATACC	Screening for pCEV-G3 and pCEVG4 constructs
SFB018	GGATATGTATATGGTGGTAATGCC	Screening for pCEV-G3 constructs
SFB017	GAGACGATATATGCCAATACTTC	Screening for pCEV-G4 constructs

Using pCEV-G2-Ph and pCEV-G2-Km as base plasmids, a second set of plasmids where the *PGK1* promoter was replaced with the strong galactose-inducible *GAL10* promoter or the *HXT7* promoter (which is induced at low glucose concentrations) was also constructed. The complete *GAL10-GAL1* promoter region was amplified from *S. cerevisiae* S288C using primers GAL10LacF and Gal10LacR (Table 3). The pSF011 promoterless target vector was opened using BamHI and XbaI restriction sites and the amplified fragment with homologous arms was cloned in by homologous recombination as described previously [64] using *E. coli* strain One Shot Omnimax 2 T1 (Life Technologies). The resulting plasmid was called pGAL10Lac. The *GAL10* promoter was then PCR-amplified from pGAL10Lac using the primers GAL10P2B and GAL10P1A (Table 3). The *HXT7* promoter was PCR amplified from pSF015 [22] using the primers HXT7P1B and HXT7P2A (Table 3). The PCR products and the vectors pCEV-G2-Km and pCEV-G2-Ph were digested with FseI and SpeI; *Dpn*I was also included in the PCR fragment digests to eliminate template DNA. The vectors were purified by gel extraction and the PCR products were purified through a column (QIAquick PCR purification kit, Qiagen). Ligation products were transformed into *E. coli* α-Select Silver Efficiency chemically competent cells (Bioline). Selection was performed on ampicillin (100 μg/mL). Colonies were screened by PCR using ADH1-T R1 paired with either SFB018 for pCEV-G3 constructs or SFB017 for pCEV-G4 constructs (Table 3). Plasmids were confirmed by restriction analysis and sequencing. The resulting plasmids were pCEV-G3-Km, pCEV-G3-Ph, pCEV-G4-Km and pCEV-G4-Ph.

The promoter:lacZ integrative plasmids pSF015 (*HXT7* promoter), pSF016 (*PGK1* promoter), and pSF019 (*TEF1* promoter), as well as the promoterless lacZ control integrative plasmid pSF011, were kindly provided by Prof. Jens Nielsen (Chalmers University of Technology, Sweden). These constructs have been described previously [22]. The Partow et al. (2010) study also included *GAL1* and *GAL10* promoters isolated from *S. cerevisiae* CEN.PK; we reconstructed the P_*GAL10*_:lacZ fusion construct by amplifying the relevant regions from *S. cerevisiae* S288C genomic DNA using GAL10LacF and GAL10LacR primers (Table 3). The pSF011 plasmid was opened using *Bam*HI and *Xba*I restriction sites and amplified fragments with homologous arms were cloned in by homologous recombination as described previously [64] using *E. coli* strain One Shot Omnimax 2 T1 (Life Technologies). The *GAL* promoter region was cloned as a complete region (P_*GAL1*_ + P_*GAL10*_ in divergent orientations) and inserted in the *GAL10* orientation to produce pGAL10Lac.

### Strain construction

*S. cerevisiae* transformation was performed using the lithium acetate method [20]. For YEp dominant marker plasmids, transformants were selected on YPD supplemented with 200 μg/mL G418 or 20 μg/mL phleomycin after a three hour pre-incubation period on non-selective medium. Integrative plasmids were linearized using NcoI prior to transformation. Selection was performed on uracil SD drop-out agar (Sigma-Aldrich; Sydney, Australia) supplemented with 2% glucose. For all transformations, biological replicates were generated as described previously [65]; briefly, three individual colonies were selected from the transformation plates and denoted as biological replicates for each new strain. Colony PCR was performed using primers CYC1-TR1 (GGGACCTAGACTTCAGGTTGTC) and Lac9434r (GAAGCCTGCGATGTCGGTTTC). Each biological replicate was purified by streaking out on uracil SD drop-out agar and inoculated into 5 mL SD uracil drop-out broth (Sigma-Aldrich; Sydney, Australia) supplemented with 2% glucose. Cultures were grown overnight at 30°C, 200 rpm. Glycerol stocks were prepared in 20% glycerol and stored at ^-^80°C.

For antibiotic sensitivity testing, EPY210C was transformed with pCEV-G2-Ph and pCEV-G2-Km to generate EPY210C(pCEV-G2-Ph) and EPY201C(pCEV-G2-Km), respectively. Biological replicates and glycerol stocks were produced as described above. To confirm plasmid presence, plasmid was extracted from each strain and verified by PCR.

### β-galactosidase assays

For β-galactosidase assays, glycerol stocks were streaked out on solid YPD and grown at 30°C. Single colonies (three per strain; technical replications) were inoculated into 5 mL YPD broths and cultured overnight at 30°C, 200 rpm. Overnight cultures were used to inoculate 30 mL YPDG to an OD_660_ of 0.2. Cultures were sampled at 24 hr and again at 3 days and 7 days. To extract protein, 5 mL culture was centrifuged (3000 × *g*, 4°C*,* 5 min) and the resulting pellet washed twice with ice-cold H_2_O. The cell pellet was resuspended in 1 mL extraction buffer (25 mM Tris–HCl, 1 mM DTT, 5 mM EDTA, pH 7.5) containing 20 μL of protease inhibitor cocktail (Sigma-Aldrich; Sydney, Australia) and the resulting suspension transferred to a pre-chilled 2 mL tube half filled with acid-washed glass beads (Ø = 0.5 mm). Cells were disrupted with a Mini Beadbeater-1 (Biospec Products, Bartlesville, OK, USA) using 4 x 20 s bead-beating bursts at 4800 oscillations per minute with 1 min cooling in ice-water between beatings. The disrupted cells were centrifuged (13 000 × *g*, 4°C*,* 5 min) and the supernatant collected. Protein concentration was determined by the method of Bradford [66] using a BioRad Protein Assay kit (BioRad, Gladesville, NSW, Australia) and bovine serum albumin as a standard. β-Galactosidase assays were performed using high-throughput microtitre plate method as described previously [67] with minor modifications, including use of a kinetic approach for the enzyme assay and standardisation to extract protein concentration. Briefly, 5 μL of supernatant extract was added in triplicate to microtitre plate wells containing 85 μL Z buffer [68]. The reaction was initiated by adding 10 μL 4 mg/mL *O*-nitrophenyl-β-d-galactoside (ONPG) prepared in phosphate buffer. The reaction was monitored at 37°C using a FLOUstar Omega plate-reading spectrophotometer (BMG Labtech, Mornington, VIC, Australia) for at least 30 min. The initial linear phase (normally within the first 8 min of the reaction) was used to calculate the rate. β-Galactosidase activity was expressed as ΔA_450_/min/mg protein. *S. cerevisiae* EPY210C transformed with linearised pSF011 was used as a negative control.

### Antibiotic sensitivity testing

Antibiotic sensitivity was tested using solid SD medium with the pH adjusted to either 5 or 7, supplemented with G418 at 200, 400, 600 or 800 µg/mL, or with phleomycin at 20, 40, 60, 80 or 100 µg/mL. Wild type S288C was used as a negative control, EPY210C(pCEV-G2-Ph) and EPY201C(pCEV-G2-Km) were used as representative strains bearing phleomycin and G418 resistance plasmids, respectively. Strains were streaked out on SD medium with the pH adjusted to 7; for EPY210C(pCEV-G2-Ph), the medium was supplemented with 20 µg/mL phleomycin, and for EPY201C(pCEV-G2-Km) the medium was supplemented with 200 µg/mL G418. Plates were grown for 2 d at 30°C. Single colonies from all three strains were streaked out on the various SD media described above and incubated at 30°C for 3 days to test antibiotic selection characteristics.

## Competing interests

The authors declare that they have no competing interests.

## Authors’ contributions

CV conceived the research, designed the approach, managed the project and wrote the manuscript. SB carried out the molecular work for plasmid vector construction and the antibiotic testing. YZ carried out the molecular work for promoter analysis and the β-galactosidase assays. LN participated in experimental design and project management. All authors contributed to revising the manuscript. All authors read and approved the final manuscript.

## Supplementary Material

Additional file 1 Plasmid Data Sheets. Click here for file
