# Supplementary material for: Dual gene expression cassette vectors with antibiotic selection markers for engineering in Saccharomyces cerevisiae
Source: Microb Cell Fact. 2013 Oct 25;12:96. doi: 10.1186/1475-2859-12-96 (PMC4231455; doi:10.1186/1475-2859-12-96)
Supplement: Additional file 1 — Plasmid Data Sheets. [file 1475-2859-12-96-S1.pdf]

pCEV-G1-Km Plasmid Features

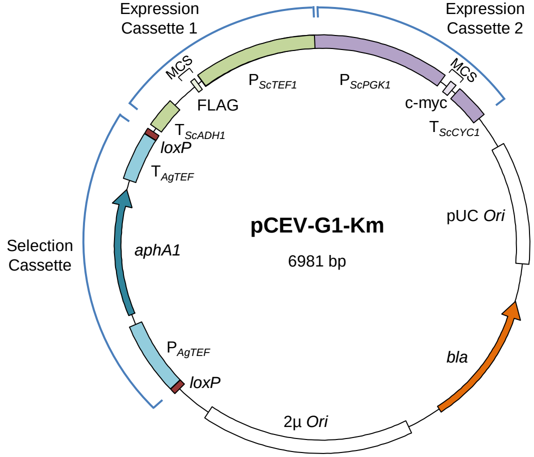

Feature	Start	End	Description
ADH1-T R1	1	21	Primer site for sequencing *TEF1* promoter fusions
T_{ScADH1}	166	2	*ADH1* terminator from *Saccharomyces cerevisiae*
FLAG	315	341	FLAG tag for protein tagging
TEF-P F1	635	616	Primer site for sequencing *TEF1* promoter fusions
P_{ScTEF1}	778	360	*TEF1* promoter from *Saccharomyces cerevisiae* CEN.PK
P_{ScPGK1}	790	1771	*PGK1* promoter from *Saccharomyces cerevisiae* CEN.PK
PGK-P F1	1525	1545	Primer site for sequencing *PGK1* promoter fusions
c-myc	1813	1848	C-myc tag for protein tagging
T_{ScCYC1}	1875	2064	*CYC1* terminator from *Saccharomyces cerevisiae*
CYC1-T R1	1925	1904	Primer site for sequencing *PGK1* promoter fusions
pUC *Ori*	2251	2918	pUC origin of replication for *Escherichia coli*
bla	3926	3069	β-lactamase gene (ampicillin resistance)
2µ *Ori*	4060	5215	2µ origin of replication for yeast
loxP	5420	5453	Recognition sequence for Cre recombinase
P_{AgTEF}	5454	5851	*TEF* promoter from *Ashbya gossypii*
aphA1	5852	6661	Aminoglycoside 3'-phosphotransferase (G418/kanamycin/neomycin resistance)
T_{AgTEF}	6662	6926	*TEF* terminator from *Ashbya gossypii*
loxP	6927	6960	Recognition sequence for Cre recombinase

pCEV-G1-Ph Plasmid Features

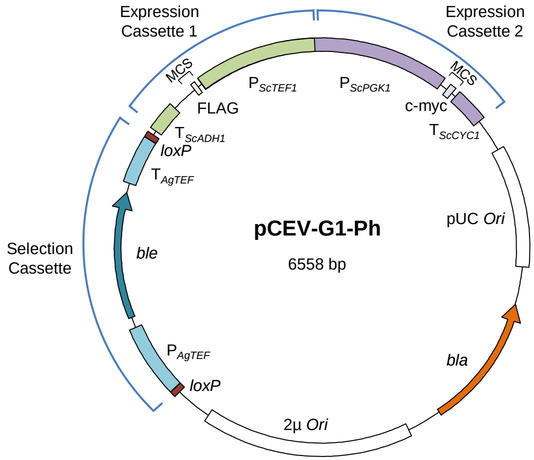

Feature	Start	End	Description
ADH1-T R1	1	21	Primer site for sequencing *TEF1* promoter fusions
T_{ScADH1}	166	2	*ADH1* terminator from *Saccharomyces cerevisiae*
FLAG	315	341	FLAG tag for protein tagging
TEF-P F1	635	616	Primer site for sequencing *TEF1* promoter fusions
P_{ScTEF1}	778	360	*TEF1* promoter from *Saccharomyces cerevisiae* CEN.PK
P_{ScPGK1}	790	1771	*PGK1* promoter from *Saccharomyces cerevisiae* CEN.PK
PGK-P F1	1525	1545	Primer site for sequencing *PGK1* promoter fusions
c-myc	1813	1848	C-myc tag for protein tagging
T_{ScCYC1}	1875	2064	*CYC1* terminator from *Saccharomyces cerevisiae*
CYC1-T R1	1925	1904	Primer site for sequencing *PGK1* promoter fusions
pUC *Ori*	2251	2918	pUC origin of replication for *Escherichia coli*
bla	3926	3069	β-lactamase gene (ampicillin resistance)
2µ *Ori*	4060	5215	2µ origin of replication for yeast
loxP	5420	5453	Recognition sequence for Cre recombinase
P_{AgTEF}	5454	5850	*TEF* promoter from *Ashbya gossypii*
ble	5852	6238	Bleomycin resistance protein
T_{AgTEF}	6245	6503	*TEF* terminator from *Ashbya gossypii*
loxP	6504	6537	Recognition sequence for Cre recombinase

pCEV-G2-Km Plasmid Features

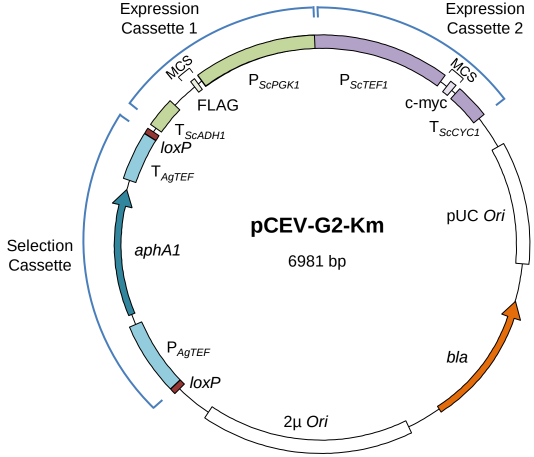

Feature	Start	End	Description
ADH1-T R1	1	21	Primer site for sequencing *PGK1* promoter fusions
T_{ScADH1}	166	2	*ADH1* terminator from *Saccharomyces cerevisiae*
FLAG	315	341	FLAG tag for protein tagging
PGK-P F1	587	607	Primer site for sequencing *PGK1* promoter fusions
P_{ScPGK1}	1343	360	*PGK1* promoter from *Saccharomyces cerevisiae* CEN.PK
P_{ScTEF1}	1354	1772	*TEF1* promoter from *Saccharomyces cerevisiae* CEN.PK
TEF-P F1	1497	1516	Primer site for sequencing *TEF1* promoter fusions
c-myc	1813	1848	C-myc tag for protein tagging
T_{ScCYC1}	1875	2064	*CYC1* terminator from *Saccharomyces cerevisiae*
CYC1-T R1	1925	1904	Primer site for sequencing *TEF1* promoter fusions
pUC *Ori*	2251	2918	pUC origin of replication for *Escherichia coli*
bla	3926	3069	β-lactamase gene (ampicillin resistance)
2µ *Ori*	4060	5206	2µ origin of replication for yeast
loxP	5420	5453	Recognition sequence for Cre recombinase
P_{AgTEF}	5454	5851	*TEF* promoter from *Ashbya gossypii*
aphA1	5852	6661	Aminoglycoside 3'-phosphotransferase (G418/kanamycin/neomycin resistance)
T_{AgTEF}	6662	6926	*TEF* terminator from *Ashbya gossypii*
loxP	6927	6960	Recognition sequence for Cre recombinase

pCEV-G2-Ph Plasmid Features

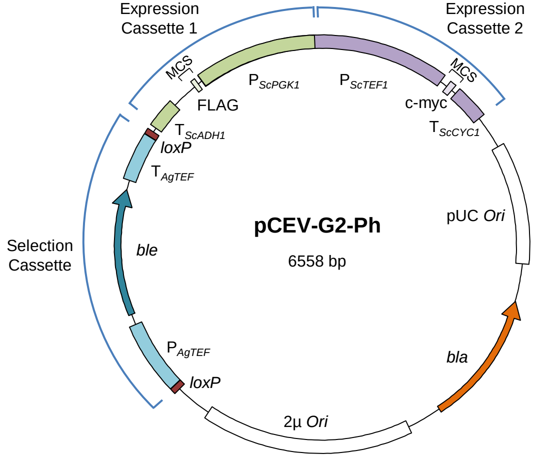

Feature	Start	End	Description
ADH1-T R1	1	21	Primer site for sequencing *PGK1* promoter fusions
T_{ScADH1}	166	2	*ADH1* terminator from *Saccharomyces cerevisiae*
FLAG	315	341	FLAG tag for protein tagging
PGK-P F1	587	607	Primer site for sequencing *PGK1* promoter fusions
P_{ScPGK1}	1343	360	*PGK1* promoter from *Saccharomyces cerevisiae* CEN.PK
P_{ScTEF1}	1354	1772	*TEF1* promoter from *Saccharomyces cerevisiae* CEN.PK
TEF-P F1	1497	1516	Primer site for sequencing *TEF1* promoter fusions
c-myc	1813	1848	C-myc tag for protein tagging
T_{ScCYC1}	1875	2064	*CYC1* terminator from *Saccharomyces cerevisiae*
CYC1-T R1	1925	1904	Primer site for sequencing *TEF1* promoter fusions
pUC *Ori*	2251	2918	pUC origin of replication for *Escherichia coli*
bla	3926	3069	β-lactamase gene (ampicillin resistance)
2µ *Ori*	4060	5206	2µ origin of replication for yeast
loxP	5420	5453	Recognition sequence for Cre recombinase
P_{AgTEF}	5454	5850	*TEF* promoter from *Ashbya gossypii*
ble	5852	6238	Bleomycin resistance protein
T_{AgTEF}	6245	6503	*TEF* terminator from *Ashbya gossypii*
loxP	6504	6537	Recognition sequence for Cre recombinase

pCEV-G3-Km Plasmid Features

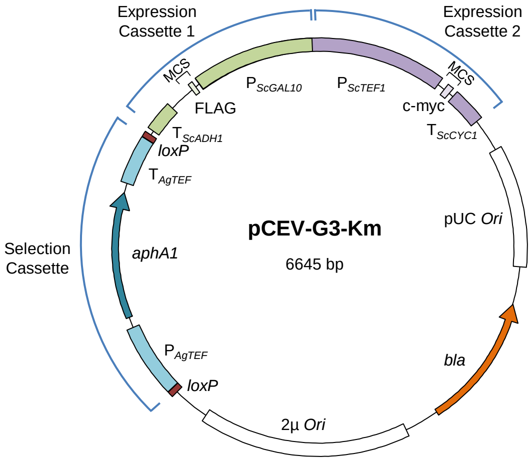

Feature	Start	End	Description
ADH1-T R1	1	21	Primer site for sequencing *GAL10* promoter fusions
T_{ScADH1}	166	2	*ADH1* terminator from *Saccharomyces cerevisiae*
FLAG	315	341	FLAG tag for protein tagging
SFB018	440	417	Primer site for sequencing *GAL10* promoter fusions
$P_{ScGAL10}$	1008	351	*GAL10* promoter from *Saccharomyces cerevisiae* CEN.PK
P_{ScTEF1}	1018	1436	*TEF1* promoter from *Saccharomyces cerevisiae* CEN.PK
TEF-P F1	1161	1180	Primer site for sequencing *TEF1* promoter fusions
c-myc	1477	1512	C-myc tag for protein tagging
T_{ScCYC1}	1539	1728	*CYC1* terminator from *Saccharomyces cerevisiae*
CYC1-T R1	1589	1568	Primer site for sequencing *TEF1* promoter fusions
pUC *Ori*	1915	2582	pUC origin of replication for *Escherichia coli*
bla	3590	2733	β-lactamase gene (ampicillin resistance)
2µ *Ori*	3724	4870	2µ origin of replication for yeast
loxP	5084	5117	Recognition sequence for Cre recombinase
P_{AgTEF}	5118	5515	*TEF* promoter from *Ashbya gossypii*
aphA1	5516	6325	Aminoglycoside 3'-phosphotransferase (G418/kanamycin/neomycin resistance)
T_{AgTEF}	6326	6590	*TEF* terminator from *Ashbya gossypii*
loxP	6591	6624	Recognition sequence for Cre recombinase

pCEV-G3-Ph Plasmid Features

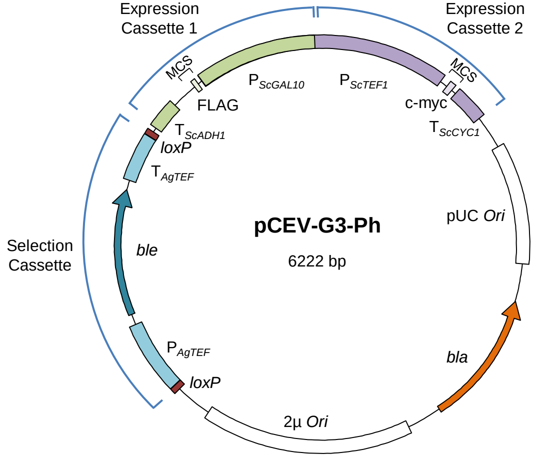

Feature	Start	End	Description
ADH1-T R1	1	21	Primer site for sequencing *GAL10* promoter fusions
T_{ScADH1}	166	2	*ADH1* terminator from *Saccharomyces cerevisiae*
FLAG	315	341	FLAG tag for protein tagging
SFB018	440	417	Primer site for sequencing *GAL10* promoter fusions
$P_{ScGAL10}$	1008	351	*GAL10* promoter from *Saccharomyces cerevisiae* CEN.PK
P_{ScTEF1}	1018	1436	*TEF1* promoter from *Saccharomyces cerevisiae* CEN.PK
TEF-P F1	1161	1180	Primer site for sequencing *TEF1* promoter fusions
c-myc	1477	1512	C-myc tag for protein tagging
T_{ScCYC1}	1539	1728	*CYC1* terminator from *Saccharomyces cerevisiae*
CYC1-T R1	1589	1568	Primer site for sequencing *TEF1* promoter fusions
pUC *Ori*	1915	2582	pUC origin of replication for *Escherichia coli*
bla	3590	2733	β-lactamase gene (ampicillin resistance)
2µ *Ori*	3724	4870	2µ origin of replication for yeast
loxP	5084	5117	Recognition sequence for Cre recombinase
P_{AgTEF}	5118	5514	*TEF* promoter from *Ashbya gossypii*
ble	5516	5902	Bleomycin resistance protein
T_{AgTEF}	5909	6167	*TEF* terminator from *Ashbya gossypii*
loxP	6168	6201	Recognition sequence for Cre recombinase

pCEV-G4-Km Plasmid Features

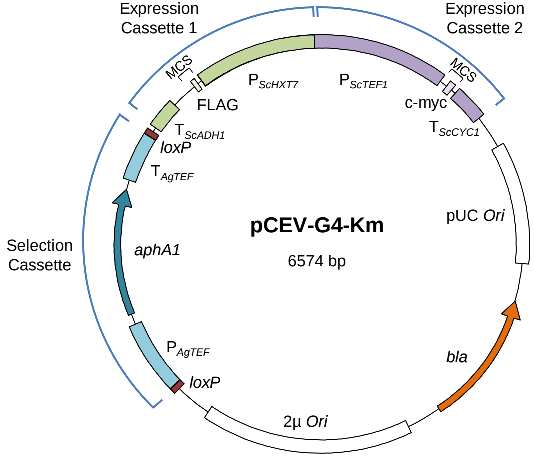

Feature	Start	End	Description
ADH1-T R1	1	21	Primer site for sequencing *HXT7* promoter fusions
T_{ScADH1}	166	2	*ADH1* terminator from *Saccharomyces cerevisiae*
FLAG	315	341	FLAG tag for protein tagging
SFB017	510	488	Primer site for sequencing *HXT7* promoter fusions
P_{ScHXT7}	937	352	*HXT7* promoter from *Saccharomyces cerevisiae*
P_{ScTEF1}	947	1365	*TEF1* promoter from *Saccharomyces cerevisiae* CEN.PK
TEF-P F1	1090	1109	Primer site for sequencing *TEF1* promoter fusions
c-myc	1406	1441	C-myc tag for protein tagging
T_{ScCYC1}	1468	1657	*CYC1* terminator from *Saccharomyces cerevisiae*
CYC1-T R1	1518	1497	Primer site for sequencing *TEF1* promoter fusions
pUC *Ori*	1844	2511	pUC origin of replication for *Escherichia coli*
bla	3519	2662	β-lactamase gene (ampicillin resistance)
2µ *Ori*	3653	4799	2µ origin of replication for yeast
loxP	5013	5046	Recognition sequence for Cre recombinase
P_{AgTEF}	5047	5444	*TEF* promoter from *Ashbya gossypii*
aphA1	5445	6254	Aminoglycoside 3'-phosphotransferase (G418/kanamycin/neomycin resistance)
T_{AgTEF}	6255	6519	*TEF* terminator from *Ashbya gossypii*
loxP	6520	6553	Recognition sequence for Cre recombinase

pCEV-G4-Ph Plasmid Features

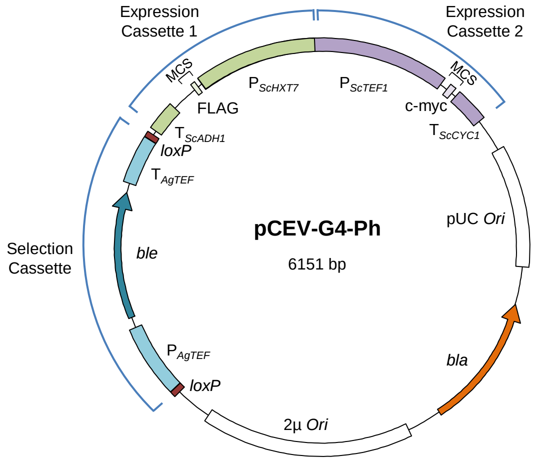

Feature	Start	End	Description
ADH1-T R1	1	21	Primer site for sequencing *HXT7* promoter fusions
T_{ScADH1}	166	2	*ADH1* terminator from *Saccharomyces cerevisiae*
FLAG	315	341	FLAG tag for protein tagging
SFB017	510	488	Primer site for sequencing *HXT7* promoter fusions
P_{ScHXT7}	937	352	*HXT7* promoter from *Saccharomyces cerevisiae*
P_{ScTEF1}	947	1365	*TEF1* promoter from *Saccharomyces cerevisiae* CEN.PK
TEF-P F1	1090	1109	Primer site for sequencing *TEF1* promoter fusions
c-myc	1406	1441	C-myc tag for protein tagging
T_{ScCYC1}	1468	1657	*CYC1* terminator from *Saccharomyces cerevisiae*
CYC1-T R1	1518	1497	Primer site for sequencing *TEF1* promoter fusions
pUC *Ori*	1844	2511	pUC origin of replication for *Escherichia coli*
bla	3519	2662	β-lactamase gene (ampicillin resistance)
2µ *Ori*	3653	4799	2µ origin of replication for yeast
loxP	5013	5046	Recognition sequence for Cre recombinase
P_{AgTEF}	5047	5443	*TEF* promoter from *Ashbya gossypii*
ble	5445	5831	Bleomycin resistance protein
T_{AgTEF}	5838	6096	*TEF* terminator from *Ashbya gossypii*
loxP	6097	6130	Recognition sequence for Cre recombinase
